# Maximizing the Utility of Transcriptomics Data in Inflammatory Skin Diseases

**DOI:** 10.3389/fimmu.2021.761890

**Published:** 2021-10-29

**Authors:** Jingni Wu, Zhixiao Fang, Teng Liu, Wei Hu, Yangjun Wu, Shengli Li

**Affiliations:** ^1^ Precision Research Center for Refractory Diseases, Institute for Clinical Research, Shanghai General Hospital, Shanghai Jiao Tong University School of Medicine, Shanghai, China; ^2^ Department of Gynecologic Oncology, Fudan University Shanghai Cancer Center, Fudan University, Shanghai, China

**Keywords:** transcriptomics, inflammatory skin diseases, RNA-Seq, bioinformatics, atopic dermatitis, psoriasis

## Abstract

Inflammatory skin diseases are induced by disorders of the host defense system of the skin, which is composed of a barrier, innate and acquired immunity, as well as the cutaneous microbiome. These disorders are characterized by recurrent cutaneous lesions and intense itch, which seriously affecting life quality of people across all ages and ethnicities. To elucidate molecular factors for typical inflammatory skin diseases (such as psoriasis and atopic dermatitis), transcriptomic profiling assays have been largely performed. Additionally, single-cell RNA sequencing (scRNA-seq) as well as spatial transcriptomic profiling have revealed multiple potential translational targets and offered guides to improve diagnosis and treatment strategies for inflammatory skin diseases. High-throughput transcriptomics data has shown unprecedented power to disclose the complex pathophysiology of inflammatory skin diseases. Here, we will summarize discoveries from transcriptomics data and discuss how to maximize the transcriptomics data to propel the development of diagnostic biomarkers and therapeutic targets in inflammatory skin diseases.

## Introduction

The skin is the outmost layer of the body. It not only acts as the first line to defense against various biotic and abiotic stresses, but also play crucial roles in water homeostatic and thermoregulation. Dysregulation of the host defense system of the skin is usually accompanied by immune-mediated inflammation and abnormal keratinocyte differentiation that subsequently induce inflammatory skin diseases. Psoriasis and atopic dermatitis (AD) are the most common chronic inflammatory skin diseases (1, 2). The pathogenesis of these two inflammatory skin diseases is complex, with disease progression driven by a combination of multiple factors, including environmental factors, genetic factors in skin barriers, dysbiosis of skin resident microbiomes, and immune system defects (3–5). Various cutaneous cellular changes, like T-lymphocyte infiltration, vascular hyperplasia can be found in infected skin of psoriasis (3, 4). AD is clinically characterized by chronic, pruritic eczematous skin lesions (5). Different subsets of TH cells, for example, TH17 and TH2/TH22 for psoriasis and AD, respectively, trigger different arrays of cytokines (6). Although both psoriasis and AD can occur at any age, AD prefers to affect infants, especially those at 3-6 months old (5), while the peak onset of psoriasis is in adolescence and early adulthood (7).

Over the past decade, high-throughput RNA-sequencing (RNA-seq) has proven to be an indispensable tool for transcriptome-wide analysis of transcriptional variations. Various types of RNAs, including messenger RNAs (mRNAs), microRNAs (miRNAs), long noncoding RNAs (lncRNAs) and circular RNAs (circRNAs), can be captured by RNA-seq (8). Therefore, broad applications of RNA-seq open a window for deep understanding of multiple aspects of molecular biology in inflammatory skin diseases, such as mRNA splicing and non-coding RNAs regulation (9, 10). Among main RNA-seq technologies, short-read sequencing of cDNA comprises the majority of current available RNA-seq data of inflammatory skin disease studies. Questions that when and where transcription occurs and how transcription is regulated are intensively studied. Recently, newly developed single-cell and spatial transcriptomic sequencing are emerging as powerful techniques for mapping and quantifying transcriptional activity at single-cell and spatial resolution. These powerful techniques have been widely and successfully used in humans, animals and plants to resolve intercellular transcriptomics heterogeneity at single-cell level (11–13). Using scRNA-seq, exciting insights about the unique characteristics of skin-resident innate lymphoid cells, key inflammatory pathways as well as inflammatory fibroblasts have been reported (14–16).

In this review, we will summarize perspectives from the big data view to characterize the current understanding of inflammatory skin diseases, specially focusing on common and bias induced in transcriptional and post-transcriptional levels in psoriasis and AD (**Table 1**). These high-throughput transcriptomics studies will help to provide insightful knowledge for a more comprehensive understanding of molecular mechanisms underlying disease occurrence and pave the way to the identification of therapeutic targets for more specific and safer modulation of inflammatory skin diseases.

**Table 1:** Deregulated genes in AD and Psoriasis.

Gene	Pathways	Key Message	Disease	Ref.
IL-17	IL-17 Pathway	Central in the psoriatic pathogenesis	Psoriasis	(17, 18)
IL-17RA			Psoriasis	(17–19)
IL-17F			Psoriasis	(17, 18)
IL-12	IL-12 Pathway		Psoriasis	(17–20)
IL-12B			Psoriasis	(17, 18)
IL-22	IL-22 Pathway		Psoriasis	(17, 18)
IL-23	IL-23 Pathway		Psoriasis	(17, 18)
IL-6	IL-17 mediated inflammatory Pathway		Psoriasis	(17)
IL-1α			Psoriasis	(17, 21)
CXCL1	Immunomodulatory chemokines		Psoriasis; AD	(17, 22)
CCL20			Psoriasis	(17)
IL-1α	IL-1 pathways	Mayor epidermal proinflammatory cytokines and positively correlated with symptoms in psoriasis and AD		(17, 21)
IL-36		Function on both fibroblasts and KCs, as well as immune system	Psoriasis; AD	(21, 23)
TNF-α	TNF-α pathway	An important inflammatory cytokine secreted by macrophages. Functions in active DCs to induce IL-23 secretion, and activate KCs to induce KRT6 and KRT16.	Psoriasis	(21)
IL-8	KC-derived inflammatory mediators	Plays a causative role in acute inflammation	Psoriasis	(24)
IL-9	TH9 cytokine	Enhancing cytokine secretion from TH1, TH2 and TH17 cells to amplify immune responses	Psoriasis	(24)
IL-20	KC-derived inflammatory mediators	Function in promote cutaneous inflammation	Psoriasis	(24)
IL-24	IL-20 family cytokine	Binging with IL-20 receptors to activates JAKs and STAT3 signaling pathway	Psoriasis; AD	(25, 26)
IL-13	TH2 cytokine	Central in AD pathogenesis; Also reported to be elevated in psoriasis	Psoriasis; AD	(25, 27, 28)
TGF-α		Function as immune-suppressor	Psoriasis	(25)
IFN-γ	IFN-γ Pathway	Induce CXCL10 and CXCL11 in KCs	Psoriasis	(25)
IL-31	IL-31 Pathway	Key factor to trigger itch	Psoriasis; AD	(25, 29)
TRPV1	Calcium-permeable cation TRPs channels	Cross talk with neurons and immune responses	Psoriasis; AD	
TRPVM8			Psoriasis	(30)
TRPV3			Psoriasis	(30)
TRPC4			Psoriasis	(30)
IL-4	TH2 type inflammation loop	Central in AD pathogenesis	AD	(27, 28, 31, 32)
IL-5		Essential eosinophil growth factor, function in differentiation.	AD	(31, 32)
RAD50		An important DNA repair molecule; Effect IgE regulation	AD	(5)
IgE		Genetic marker of AD	AD	(32)
TSLP	Type 2 inflammatory cytokines	Genetic marker of AD	AD	(33)
IL-33		Sufficient for AD development; Induce IL-31 to promote itch; Reduce filaggrin and claudin-1	AD	(34)
IL-25		Important in regulation of skin inflammation	AD	(35)
TREM-1	Innate and adaptive immunity	A neutrophils expressed receptor, function in pattern recognition	AD	(23)
IL-10	IL-10 family cytokines	An anti-inflammatory cytokine, central in infection by limiting immune responses	AD	(22)
KRT16	Epidermal differentiation pathway	Functions on epidermal differentiation	AD	(22)
S100A8		Function as a Ca^2+^ sensor, and important in modulating the inflammatory response	AD	(22)
S100A9			AD	(22)
CXCL6	Immunomodulatory chemokines	Up regulated by IL-4	AD	(22)
FOXK1	Negative regulator of T-cell activation	Act as immune regulator	AD	(22)
FLG	Epidermal differentiation complex	Function in maintenance of skin barrier	AD	(22)
LOR			AD	(22)
KRT10			AD	(22)
KLK5	Kallikren related peptidase	Degrading desmosomal proteins and inducing proinflammatory cytokine secretion *via* protease activity	AD	(29)
KLK14			AD	(29)
KLK7			AD	(36)
SPINK5	Protease inhibitors	Regulate epidermal differentiation *via* Wnt-beta-catenin Pathway	AD	(29)
AQP3	Aquaporin	Regulate epidermal water homeostasis	AD	(29)
TRPV2	Calcium-permeable cation TRPs channels	Cross talk with neurons and immune responses	AD	(30)
TRPA1			AD	(30)

## Deep Understanding of Inflammatory Skin Diseases From Large-Scale Transcriptomics Data

### Best Practice for Excavating Molecular Treasures From RNA-Seq Data

**Figure 1:**
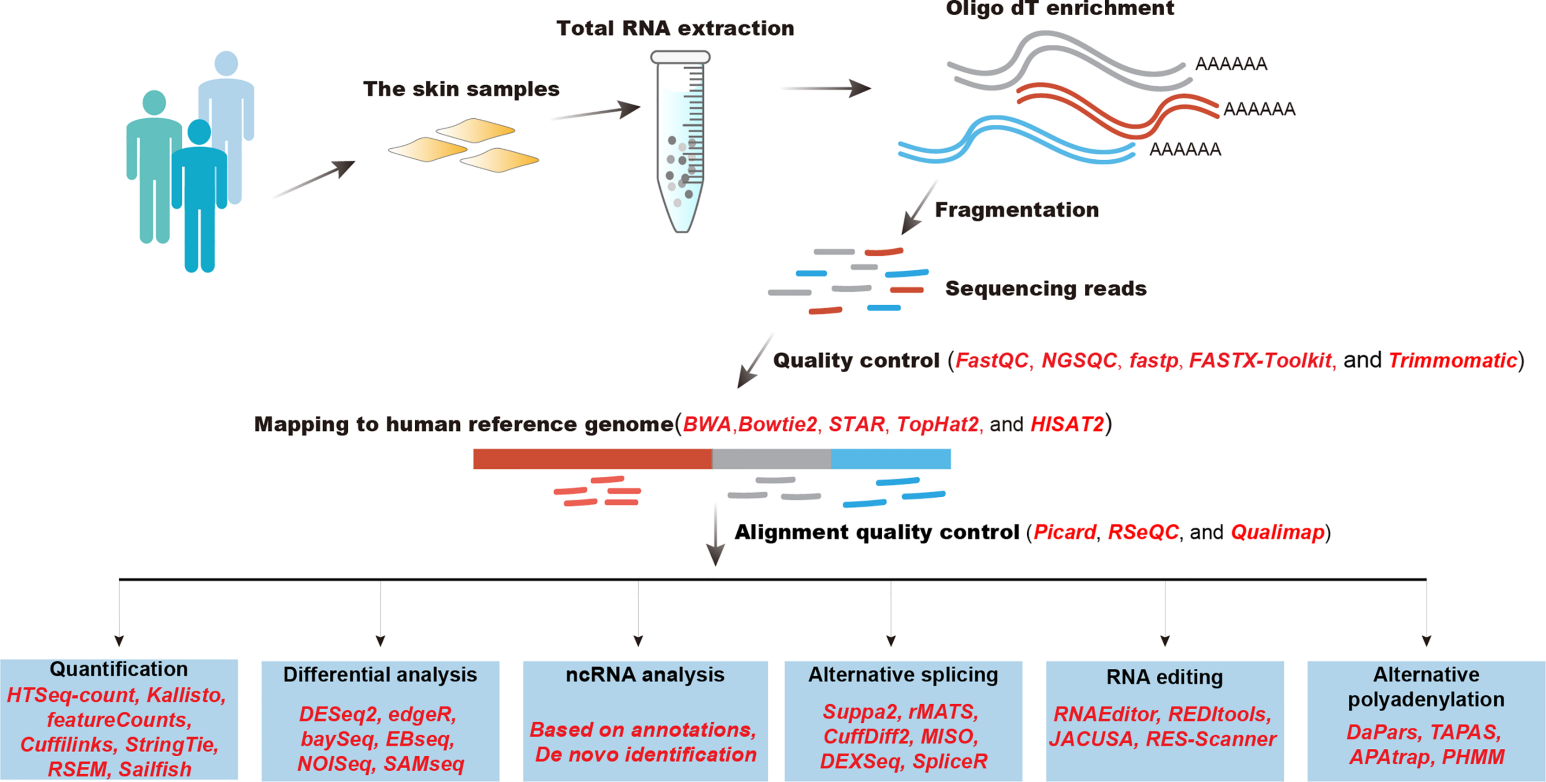
A best practice of RNA-seq data analysis of inflammatory diseases.

The rapid development of computational algorithms has largely expanded our understanding of molecular mechanisms of inflammatory skin diseases. After obtaining sequencing reads derived from human skin samples, quality control should be performed first (**Figure 1**). Computational tools, such as FastQC, NGSQC (37), fastp (38), FASTX-Toolkit, and Trimmomatic (39), can be used to evaluate the quality of sequencing bases and trim low-quality bases and reads. Next, quality-controlled RNA-seq reads are mapped to the human reference genome to determine where they are from by using such aligners as BWA (40), Bowtie2 (41), STAR (42), TopHat2 (43), and HISAT2 (44). Alignment results should be examined to filter low-quality read alignments by utilizing Picard (https://broadinstitute.github.io/picard/), RSeQC (45), or Qualimap (46). Then, reads mapped to genomic regions of specific genes were calculated to determine transcriptional abundance, this step could be realized by HTSeq-count (47), Kallisto (48), featureCounts (49), Cuffilinks (50), StringTie (51), RSEM (52), and Sailfish (53). Advanced analysis of RNA-seq data includes differential expression analysis, ncRNA analysis, alternative splicing (AS), RNA editing, and alternative polyadenylation (APA). The differential analysis is designed to statistically compare the same genes between different conditions to determine functional gene sets that participate in the development of pathological or physiological conditions. Frequently used computational tools that perform differential analysis are DESeq2 (54), edgeR (55), baySeq (56), EBseq (57), NOISeq (58), and SAMseq (59). Noncoding RNAs constitute the major component of human genome and exert important regulatory roles in a variety of physiological and pathological processes. The identification and quantification of noncoding RNAs could be performed based on the reference annotation or *do novo* transcriptome assembly (60). AS is the major contribution to transcriptional diversity in humans, which plays crucial roles in the pathological process of diseases. Computational algorithms, such as Suppa2 (61), rMATS (62), CuffDiff2 (63), MISO (64), DEXSeq (65), and spliceR (66), were designed to identify and quantify AS events and the alternative usage of gene exons. RNA editing is a post-transcriptional event that alters single bases at RNA levels, which has been demonstrated to modulate the AS process, RNA expression, and RNA translation. Multiple tools have been developed to identify RNA editing sites, such as RNAEditor (67), REDItools (68), JACUSA (69), and RES-Scanner (70). APA event is the alternative processing of mRNA 3’ end that generates mRNAs with diverse lengths of 3’ UTR.APA is emerging to play important roles in regulating RNA metabolism, which could be detected by DaPars (71), APAtrap (72), and PHMM (73). We will summarize major findings from analyzing large-scale transcriptomics data in inflammatory skin diseases.

### Keratinocyte Responses in Psoriasis and AD

Keratinocytes (KCs) were previously thought of passive bystanders in the inflammatory process. Nowadays, growing evidence has shown that KCs are actively involved in the initiation and maintenance of skin inflammation, by secreting chemokines, cytokines and antimicrobial peptides, which further amplifies the inflammatory process through chemoattraction and activation of skin immune cells (17).

In psoriasis, IL-17 activates epidermal KCs *via* IL-17R to produce proinflammatory cytokines and chemokines, such as IL-1, IL-6, CXCL1 (C-X-C motif), and CCL20 (C-C motif ligand 20) to induce and propagate psoriatic inflammation (17), while IL-23 drives a local skin inflammatory loop through IL-23 triggered production of IL-22, which in turn helps the maintenance of TH17 cells (18). Therefore, the interplay between IL-23-IL-17 axis and KCs is believed as the central of IL-17-mediated inflammatory loop in psoriasis. Thousands of differentially expressed genes (DEGs) by using large-scale transcriptome analysis were identified in psoriasis. As expected, IL-17, IL-1/IL-36, IL-12/IL-23, and TNF-α pathways were revealed to be the top enriched pathways (19, 21, 24, 74). To further reveal the cellular and molecular mechanisms behind this, IL-17 receptor-A (IL-17RA) antagonist brodalumab, was applied in moderate and severe psoriasis over 12 weeks. This treatment-induced global gene expression profiles revealed a rapid and extensive suppression of IL-17-dependent genes in KCs as well as specific KC-derived inflammatory mediators such as IL-8, IL-9, and IL-20 (24). Compared to human monoclonal antibody ustekinumab, this suppression occurs earlier and to a greater extent, suggesting that application of IL-17 antagonists producing a greater modulation of the synergistic/additive gene set of psoriasis. Similarly, IL17-RA, IL-12/IL-23, or TNF inhibition treatments, were found to mainly affect the expression of IL-17 dependent genes, which further confirmed the hypothesis that IL-17 -stimulated KCs were key drivers of psoriasis (19, 74). Page et al. reported that transcriptional dysregulation in moderate-to-severe psoriasis is dominated in IL-17 related genes in KCs. Down-regulation of IL-17A, IL-17F and IL-12B were detected as early as 2 weeks post-treatment with PF-06700841, suggesting the requirement of TYK2 and Janus kinase 1 for psoriasis signaling transduction (20). IL-36, another important mediator of psoriasis, was thought to regulate psoriasis by activating MAPKs and NF-kB pathways (75). To identify novel factors in psoriasis, RNA-seq analysis was applied in time-dependent IL-1B, IL-36A, IL-36B or IL-36G treated KC samples. This work found that early and later IL-1B-specific responses are all replicated by those of IL-36 treatments. Besides, Type I and II interferon genes exhibited a time-dependent response pattern: inducted at 8 h following no response or repression at 24 h, suggesting a fine-scale characterization of time and cytokine-specific response patterns after IL-1B and IL-36 treatment (76). Early studies showed that the inflammatory loop of AD initiates once skin-resident DCs or other immune cells are stimulated by external agents, with a consequence of releasing epithelial type 2 inflammatory cytokines, such as TSLP, IL-33, and IL-25 (33–35). Meanwhile, KC responses can also be directly induced by external agents to affect subsequent inflammatory events (18, 77). To explore molecular mechanisms behind this, RNA-seq by using biopsy specimens have been largely performed (22, 23, 29, 78). The first study of lesional AD RNA-seq, reported that TREM-1 and IL-36 were novel factors of AD (23). Recently, Tsoi and colleagues showed that dysregulated genes accompanying the transition from nonlesional to acute and chronic AD quantitatively different. Enrichment of DEGs in TNF, TH1, TH2, and TH17 pathways was observed during the whole process of disease progression in nonlesional, acute, and chronic AD. And the most heightened inflammatory response in chronic AD samples. Besides, 42 significant dysregulated genes involving in epidermal differentiation (e.g., IL-10, *KRT16*, *S100A8*, and *S100A9*), antimicrobial, immunomodulatory chemokines (CXCL1, CXCL6), and negative regulation of T-cell activation (FOXK1) were found in chronic versus acute AD (22). These findings provide novel insights and highlight underappreciated pathways in AD pathogenesis that may amenable to be future therapeutic targets. Medical treatment of moderate-to-severe AD-like oral JAK/SYK inhibitor ASN002 induces rapid and sustained improvements in TH2, TH22/TH17, and TH1 pathways, as well as epidermal barrier abnormalities (78). AD in pediatric and Asian populations trigger strong TH2 and TH17 responses, whereas European American adults mainly induce TH2/TH22 activation, suggesting diverse oral therapeutics in treatment with moderate-to-severe AD in different region patients need to be carefully chosen. Very recently, study exploring moderate-to-severe AD before and after systemic treatment characterized a “core” signature of AD by dysregulation of genes related to keratinocyte differentiation and IL-31/IL-1 itch signaling. Additionally, a dynamic signature reflects progressive immune response dominated by type 2 cytokines, with an additional role of TH17 and natural killer cell signaling (29).

### Skin Barriers in Psoriasis and AD

The skin can be mainly divided into three layers: epidermis (consists of four strata of keratinocytes), dermis (mainly consists of fibroblasts and immune cells), and subcutaneous layer (consists of subcutaneous fat and connective tissue) (79). As a complex and dynamic system, skin barriers consist of four parts: microbiome barrier (skin microbiome), chemical barrier (stratum corneum), physiological barrier (mainly by epidermis), and immune barrier (epidermis and dermis) (80).

Stratum corneum and tight junctions (TJs) in granular cells are two main constituents of the physiological barrier (81). IL-17 was found to downregulate filaggrin expression and impaired TJs of the skin (82). Though the role of Langerhans cells (LCs) in psoriasis pathogenesis is still controversial, it seems like that inflammation-associated LCs in impaired psoriasis barrier gain an enhanced capacity to promote polarization of naïve T cells into TH17, and subsequently induce TH17 responses (83).

As showed by Swindell. *et al*, a large number of psoriasis-induced DEGs were also differentially expressed in other immune defective diseases and epidermal differentiation complex (EDC), reflecting intrinsic immune defects in psoriatic KCs may contribute to compromising barrier function (84). To achieve a more comprehensive understanding of molecular pathogenesis, Zhang *et al.*, re-analysis the gene expression profiles of 175 pairs of lesional and non-lesional skin samples from 5 previously published datasets. In this work, they showed that the most enriched biological processes of DEGs in psoriasis samples were immune responses and epidermal differentiation/development (85). In another study, 127 DEGs were screened and the most enriched GOs were keratinization by in-depth bioinformatic analyses of datasets deposited in GEO (GSE13355 and GSE14905) (86). Taking all these together, physical barrier is speculated to be critical for the development of psoriasis.

As defects in either stratum or tight junction triggers TH2 response, the crucial role of skin barriers in the development of AD was well accepted (87). Key epidermal genes, such as filaggrin (*FLG*), loricrin (*LOR*), and *KRT10*, which are also known as EDC genes, were frequently dysregulated in AD (88–90). Downregulation of these EDC genes that function as a physical barrier or antimicrobe factors were proposed to contribute to AD pathogenesis. Evidence supporting this is that mutation of the epidermal structure protein FLG causes approximately 20-40% of AD (5). Besides epidermal disturbances in structural proteins, proteases and their inhibitors are commonly the top dysregulated genes in AD transcriptomic profiles (29). This might be due to the maintenance of skin barrier function largely relies on homeostatic conditions between cellular motility and excessive tissue destruction. Kallikrein-related peptidase (KLK) genes, such as *KLK5*, *KLK14*, and lipid metabolism gene *ELOVL1*, were down-regulated in AD skin (29), while protease inhibitor *SPINK5* (serine protease inhibitor of kazal type 5) and human aquaporin 3 (AQP3) were up-regulated, which function in elevating inflammatory responses and epidermal water loss, respectively (29, 91).

### Sensory Nerves in Psoriasis and AD

Since all itch stimuli sensed in the cutaneous are ultimately transmitted through nerves to the brain, the crucial role of the nervous system is thought as key factor to understand the mechanisms underlying itch in inflammatory skin diseases. Neuropeptides like substance P (SP) and calcitonin Gene-Related Peptides (CGRPs) were reported to be important in the enhancement of itch in psoriasis (92, 93). TRP cation channels are a superfamily of nonselective calcium-permeable cation channels, that in coupling with pruritogenic CGRPs (94, 95). TRPs such as TRPV1, TRPM8, TRPV3 and TRPC4 were reported to be significantly elevated in pruritic skin with psoriasis (30, 96). Meanwhile, various immune cells, such as T cells or mast cells, secrete diverse cytokines that directly or indirectly aggravate itch by increasing inflammatory responses. Examples are the well-known TH17 and TH12 cytokines IL-17, IL-22 and IL-23 (97, 98). Besides, nociceptors were also found to interact with dermal dendritic cells (DDCs) and regulate IL-23/IL-17 pathway *via* TRPV1 and Nav1.8 (99). In addition, gene transcription of IL-31 was elevated in pruritic lesions of psoriasis (30). While application Janus Kinase-Signal Transducer and Activator of Transcription (JAK-STAT) inhibitor down-regulating mRNA expression levels of IL-22, IL-23, and IL-31 to effectively attenuate itch in psoriatic patients (100–102). These studies highlighted the crosstalk between peripheral nerves and immune system in the development of psoriasis.

Similar to psoriasis, chronic itch is a well-defined symptom of AD. Individual proprioceptors are defined to respond to specific pruritogens, such as non-histaminergic to induce chronic itch in AD (103). Recently, the role of IgE-basophil-leukotrienes (LT) were revealed in the activation AD-caused acute itch flares (104). Likely, TH2 lymphocytes, eosinophils, neutrophils and mast cells work together to amplify inflammatory and itch pathways *via* releasing cytokines and neurogenic peptides (105, 106). Some AD-associated pro-inflammatory TH2 cytokines, such as IL-4 and IL-13 can directly activate sensory neurons through JAK signaling pathways (27). Additionally, IL-31 and TSLP also directly interact with cation channel TRPV1^+^TRPA1^+^ neurons to trigger robust itch behaviors in AD (107, 108). RNA-seq analysis showed that serine proteases KLK7 was the most abundant and differentially expressed KLKs in both human AD and murine AD-like skin. Surprisingly, KLK7 promotes AD-associated itch independently with skin inflammation (36).

Comparative analysis of global gene regulation in paired itchy, lesional and noitchy, and nonlesional skin biopsies from AD and psoriasis identified nearly 2,000 DEGs. Among them, up-regulation of phospholipase A2 IVD, voltage-gated sodium channel 1.7, and TRPV1 were positively correlated with itch intensity in both diseases. While upregulation of TRPV2, TRPA1, protease-activated receptor 2, protease-activated receptor 4, and IL-10 were only detected in AD, and TRPM8, TRPV3, phospholipase C and IL-36 were psoriatic specific. This “itchscriptome” extended our understanding of the molecular mechanism of sensory nerves in inflammatory skin diseases and provide potential targets for itch treatment (30).

### Skin Microbiota in Psoriasis and AD

Due to the complexity of skin surface microenvironments, skin located microbiota is quite diverse. Surveys of discrete skin sites, that were selected from microbial infections sites, demonstrated that skin physiology (moist, dry, and sebaceous) is the result of organized skin microbiota communities (109). Skin microbes metabolized host proteins and lipids to release bioactive molecules. These skin microbe-released antimicrobial peptides or metabolites can be directly coupled by host skin-resident dendritic cells (DCs) and trigger host immune responses. Meanwhile, these molecules released from skin microbiota promote host to secret cytokines (such as IL-1, IL-17) and further enhance cell microbicidal function (109). Dysbiosis of skin microbiota therefore is highly associated with inflammatory skin disease.

To reveal the role of microbiota in inflammatory skin diseases, large-scale analysis using 16S and RNA-sequencing have been performed and presented distinct microbiome compositions in AD and psoriasis skin. Unlike AD, wherein only, *S. aureus* was identified as the dominant microbe, multiple organisms including *Corynebacterium* and *Finegoldia* were identified in psoriasis skin. *Corynebacterium* was considered to negatively regulate interferon signaling to affect the initiation of psoriasis (110), while colonization or infection of bacterium *S. aureus* has been frequently reported in AD (111). Using 16S and RNA-sequencing of AD samples, a significant increase of *S. aureus* and loss of anerobic species, accompanying with host response by altering expression of genes related to barrier function, metabolic reprogramming, antimicrobial defense, and TH2 signaling was detected (110).

## Discoveries of Dysregulated Noncoding RNAs in Inflammatory Skin Diseases

Besides protein-coding RNAs, the human genome also transcribes a large number of noncoding RNAs that have been classified into multiple families based on their size and biogenesis (112). These noncoding RNAs have been revealed to play pivotal roles in human complex diseases including cancer, autoimmunity diseases, and hereditary diseases (113–115). And deep mining of noncoding RNA transcriptomic data will provide the opportunity to discover novel biomarkers and therapeutic targets in inflammatory skin diseases (**Table 2**) (8).

**Table 2:** Noncoding RNAs in AD and Psoriasis.

Noncoding RNAs	Targets/Regulators	Key Message	Disease	Ref.
miR-203	SOC3; NR1H3/LXR-α; PRAR-ϒ	Regulate JAK2/STAT3 signaling pathway, by direct targeting SOC3; Modulated the proliferation of keratinocytes through direct targeting to LXR-α/PRAR-ϒ	Psoriasis; AD	(116–119)
miR-146a	CCL5; TRAF6; IRAK1; CARD10; COPS8	Negatively regulated keratinocyte proliferation and inflammation pathways by targeting CCL5, TRAF6, IRAK1; Inhibits GRCR-mediated NF-ƘB activity by targeting CARD10 and COPS8	Psoriasis	(120–122)
miR-155	CTL4; PKIα; GATA3	Associated in keratinocyte proliferation, inflammation and tight junction disruption; In T cells, it promotes T cell proliferation and TH17 responses by directly targeting CTL4; Regulate GATA3 and IL-37 mediated inflammatory responses in Psoriasis	AD; Psoriasis	(123–125)
miR-223	PTEM	Positively correlation with Treg cell; Involved in AD through indirectly upregulating HMT to degrade excessive histamine in AD; Increase proliferation and inhibited apoptosis in IL-22 stimulated KCs	Psoriasis; AD	(126–129)
miR-143	IL-13Rα1	Decrease IL-13 activity and inflammatory responses *via* targeting IL-13Ra1 in AD;	Psoriasis; AD	(126, 127)
miR-369-3p	iNOS	miR-369-3p reduce NO production by targeting iNOS, and decreased the release of TNFα, IL-6, IL-12 et al. to regulate chronic inflammatory response	Psoriasis	(130, 131)
miR-151a	IL12RB2	Negatively regulate IL-12 signaling	AD	(132, 133)
lncRNA-H19	miR-130b-3p- Dsg1 axis	Regulate Dsg1 expression and consequently regulates keratinocyte differentiation through directly binds to miR-130b-3p	Psoriasis	(134, 135)
lncRNA-MSX2P1	miR-6731-5p-S100A7 axis	Up-regulation of lncRNA-MSX2P1 promotes the IL-22-stimulated keratinocytes by inhibiting miR-6731-5p	Psoriasis	(136)
lncRNA-MEG3	miR-21-caspase-8 axis	Down-regulated lncRNA MEG3 activates apoptosis though miR-21 suppressed caspase-8	Psoriasis	(137)
lncRNA PRINS	G1P3	Up-regulated lncRNA PRINS targets the anti-apoptotic G1P3 in keratinocytes, therefore diminish sensitivity of keratinocytes to spontaneous apoptosis through G1P3	Psoriasis	(138)
IncRNA SPRR2C	miR-330-STAT1-S100A7 axis	Competed with STAT1 and S1000A7 to counteract miR-330-mediated suppression of STAT1 and S100A7	Psoriasis	(139)
lncRNA MIR155HG	encode miPEP155	Encode a micropeptide to regulate antigen presentation and suppress autoimmune inflammation	Psoriasis	(140)

### miRNA

miRNAs are small (approximately 22 nucleotides) and evolutionarily conserved noncoding RNAs that regulate gene expression at the post-transcriptionally level. To date, more than 2,500 human miRNAs have been reported, and their expression levels vary significantly depending on tissue and cell types (141). Genetical evidences form both gain and loss of function approaches exhibited that miRNAs functions to regulate gene expression in at least two aspects: prepress target mRNAs and buffer posttranscriptional genetic noise (142).

Evidence is rapidly accumulating for the role of miRNAs in the pathogenesis of inflammatory skin disorders. miR-203 was the first reported keratinocyte-derived miRNA, which was shown to target SOC3, NR1H3/LXR-α and PRAR-ϒ (116–118). miR-203 was reported to be upregulated in serum but downregulated in urine, suggesting as a potential biomarker for children AD (119). Some miRNAs, such as miR-146a and miR-155, were elevated in both psoriasis and AD (120, 143). miR-146a was found to negatively regulate keratinocyte proliferation and inflammation pathways by targeting CCL5, TRAF6, IRAK1, and CARD10 (120–122), while miR-155 was demonstrated to be involved in keratinocyte proliferation, inflammation, and tight junction disruption, by directly targeting CTL4 in T cells (123–125). In psoriasis, miR155 was also reported to regulate GATA3 downstream IL-37 mediated inflammatory responses (125). Several miRNAs have been demonstrated to be efficiency diagnostic biomarkers for inflammatory skin diseases, such as miR-223, miR-143, and miR-369-3p in psoriasis (126, 130). In addition, miR-143 suppresses IL-13 activity and inflammatory responses *via* directly targeting IL-13Rα1 (127). In serum of AD patients,miR-614, miR-223, and miR-151a were significantly up-regulated, Collectively these studies suggested miRNAs as new diagnostic biomarkers for inflammatory skin diseases (128, 132, 133).

### LncRNA

LncRNAs are RNA transcripts that are over 200 nucleotides long and commonly recognized to have limited potential to encode any identifiable peptide products (144). LncRNAs can be classified into sense, antisense transcripts, long intergenic noncoding RNAs (lincRNAs), and long intronic RNAs (lncRNAs) based on their genome location (145), and can also be classified as *cis*- and *trans*-acting lncRNAs according to their function (146). Expression of lncRNA is usually tissue-restricted, development-regulated and vary largely under different disease conditions. Many studies revealed that lncRNAs may serve as scaffolds to form ribonucleoprotien (RNP) complexes or as decoys for proteins and miRNAs (147, 148).

With the widespread applications of next-generation sequencing (NGS) technologies, huge numerous of human lncRNAs have been identified, producing a plenty of lncRNA resources in different contexts, such as LNCipedia (149) and NONCODE (150). Recently, an enrichment of dysregulated lncRNAs of typical inflammatory skin diseases has been reported (151). These dysregulated lncRNAs were mainly involved in epidermal differentiation, apoptosis and immune responses pathways (134, 140, 152). For example, lncRNA-H19 regulate Dsg1 expression and consequently regulates keratinocyte differentiation through directly binding to miR-130b-3p (135). Similarly, Qiao et al. reported that the expression level of lncRNA-MSX2P1 positively correlated with S100A7. And up-regulation of lncRNA-MSX2P1 promoted the IL-22-stimulated KCs by inhibiting miR-6731-5p, suggesting a network module of lncRNA-MSX2P1-miR-6731-5p-S100A7 (136). By using weighted gene co-expression network analysis (WGCNA), 67 miRNA-lncRNA co-expression pairs have been found (153). Studies of lncRNA SPRR2C revealed that it competed with STAT1 and S1000A7 to counteract miR-330-mediated suppression of STAT1 and S100A7 (139). Psoriatic down-regulated lncRNA MEG3 was found to activate apoptosis through miR-21-suppressed caspase-8 (137). Alternatively, psoriatic up-regulated lncRNA PRINS targets the anti-apoptotic G1P3 in KCs, therefore diminishing the sensitivity of KCs to spontaneous apoptosis through G1P3 (138). Recently, several lncRNAs that were previously annotated as noncoding RNAs are reported to encode micropeptides or small proteins (140, 154, 155). These lncRNA-encoded micropeptides were shown to be key regulators of vital cell functions, such as muscle development, cancer development, and inflammatory skin disease development. Niu et al. found that lncRNA MIR155HG could encode a micropeptide to regulate antigen presentation and suppress autoimmune inflammation in psoriasis, unlike well-known lncRNA/miRNA module, this study, for the first time, illustrated a new mechanism of lncRNA in inflammatory diseases (140).

## Post-Transcriptional Regulation in Inflammatory Skin Diseases

Applications of NGS-based methods revolutionize our understanding of dysregulated genes at not only the transcriptome level but also post-transcriptional levels. It is known that human immature transcripts go through extensive post-transcriptional regulation to generate mature functional transcripts (156). Major post-transcriptional events such as AS and RNA editing are well studied in human complex diseases, including cancer at immune diseases (157, 158). Here, we will discuss the important roles of post-transcriptional events in the progression of inflammatory skin diseases.

### Alternative Splicing

AS is a post-transcriptional process by which pre-mRNA transcripts are spliced in different ways. Nearly all human protein-coding genes undergo one or more forms of alternative splicing to generate various functional mRNA and protein products from a single gene. This process largely contributes to the complexity in the transcriptome and lead to protein diversity (8). AS needs the formation of the splicing complex to exert inclusion or skipping of exons, alternative 5’ splice-site selection, intron retention, exclusive splicing of adjacent exons, and switching between alternative splice sites (159–161). The formation of the splicing complex is a series of interplays between small nuclear ribonucleoprotein particles (snRNPs, U1, U2, U4/6, and U5), small nuclear RNAs (snRNAs), and overs 150 additional associated proteins which not directly bound to the snRNPs (162). With the growing applications of RNA-seq, more roles of AS events are unveiled. For example, cancer cells can generate cancer type-specific and subtype-specific alterations in the splicing process and contribute to cancer progression as well as cancer immune responses (163). Similarly, Shimizu et al. reported that the ST2 gene encoded both membrane-bound ST2L and soluble ST2 (sST2) by AS, wherein ST2L promoted TH2 activity with a result of dysregulation of TH1/TH2 immune balance and severe AD diseases (164). Human ACT1 undergoes AS in SNP-D10N region with a result of expressed ACT1 isoforms ACT-D19N and ACT-D10N. ACT1-D19N is fully responsive to IL-17 through interacting with Hsp90, while ACT-D10N loses this ability. Although these two isoforms are equally expressed in ACT1D10N/D10 fibroblasts, ACT1D10N/D10 T cell expressed predominantly ACT-D10N, leading to a dysregulated hyperactive TH17 response with elevated IL-17A and IL-22 expression in ACT1D10N/D10 T cells and consequently severe psoriasis (165). Kyong et al. reported that ESRP1-mediated AS of Rho GTP exchange factor ARHGEF11 was essential for epithelial tight junction (TJ) integrity in the dysregulation of skin barriers (166). Together, these studies not only illustrated the vital role of AS events in the development of inflammatory skin diseases but also revealed a potential application of mining AS events to deep our understanding of inflammatory skin diseases.

However, to our best knowledge, comparing with a large application of transcriptome analysis in the identification of novel AS events in cancer, most of the AS investigations in inflammatory skin diseases were low-throughput experiment-based, suggesting the potential applications of identification AS events through high-throughput transcriptomics in discovering novel factors in inflammatory skin diseases.

### RNA Editing

RNA editing is a post-transcriptional event that modifies single-base changes on RNA nucleotides without altering their genomic DNA (158). Thus, impaired RNA editing activity can lead to increased modulation of alternative splicing, missense codon changes, and modifications of noncoding RNAs (158, 167, 168) RNA editing has been reported as an import process that contributes to proteomic diversity in human diseases (169). In 2011, Cailin E. Joyce reported a low frequency of RNA editing in normal and psoriasis skin (170). This work was later confirmed by the Shoshana Greenberger group, as they reported that psoriasis patients demonstrated a global A-to-I RNA editing reduction in psoriatic lesions, which may account for the accumulation of double-stranded RNA (dsRNA). This process, in turn, stimulates the production of IFNs and is instrumental in triggering the initiation and progression of diseases (171). Besides global alteration, RNA editing changes were also detected in IGFBP7, COPA, and FLNA genes sites, suggesting a link of autoimmune diseases to a reduction in global RNA editing (171). These studies together suggested that RNA-editing mediated post-transcriptional regulation may be involved in the process of inflammatory skin diseases.

## Perspectives From Single-Cell Transcriptomics Data in Inflammatory Skin Diseases

**Figure 2:**
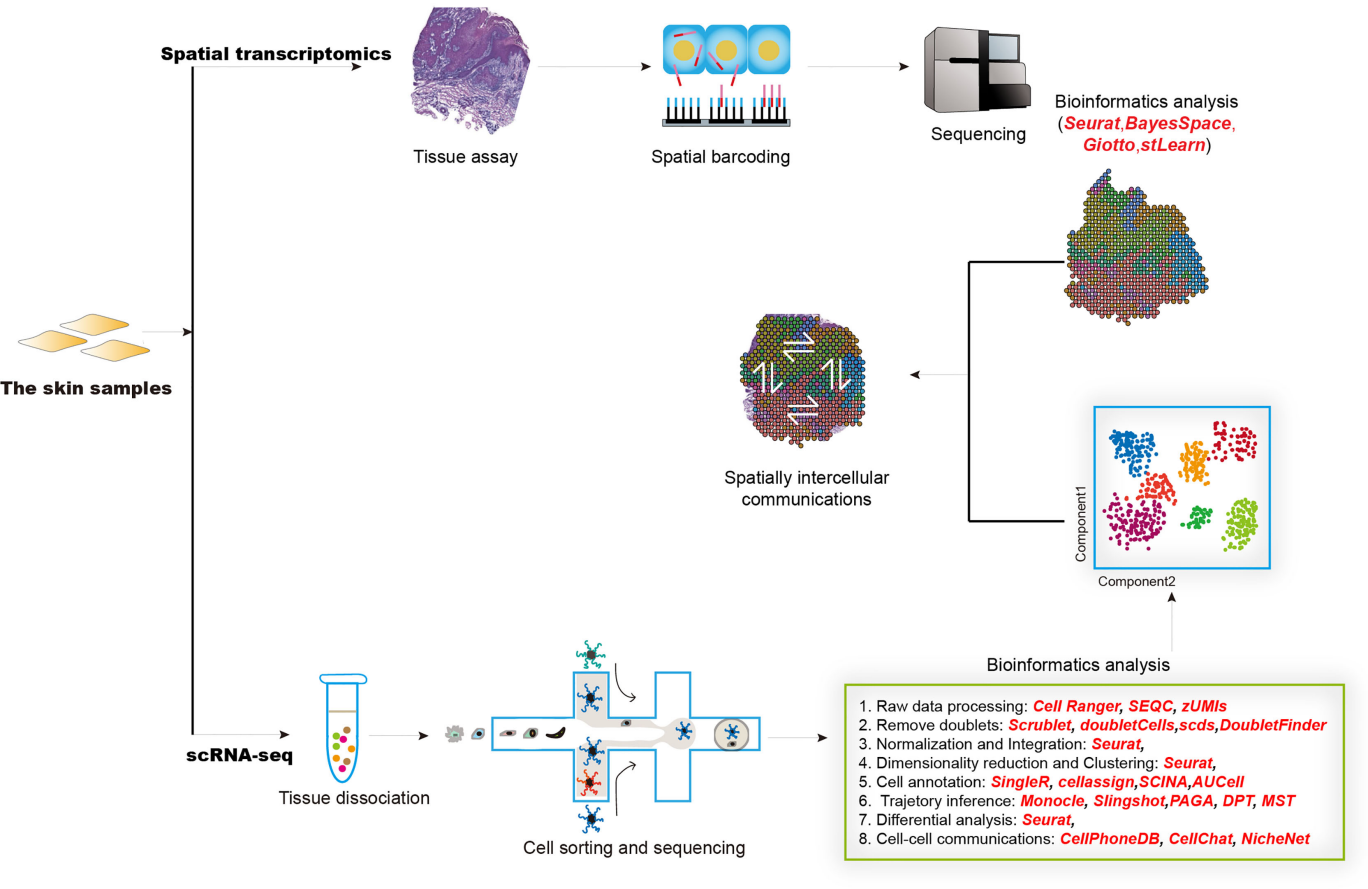
The overall design of experimental and computational analysis for single-cell transcriptomics and spatial transcriptomics studies in inflammatory diseases.

Single-cell RNA sequencing (scRNA-seq) technology is emerging as a powerful tool for characterizing heterogeneity between and within tissue/cell types. It enables more rapid identification of novel cell types, cell states, lineages as well as circuitry (172, 173). Together with spatial transcriptomics, these revolutionized techniques have been widely and successfully used in both mammalian and plant kingdoms (174, 175) and prompted our understanding of multiple complex diseases, such as inflammatory skin diseases (176). The generation of scRNA-seq data mainly includes sample collection, tissue dissociation, cell sorting, library construction, and sequencing (**Figure 2**). The raw scRNA-seq data was first mapped to reference genome by utilizing tools like Cell Ranger, SEQC (177), and zUMIs (178) to generate an expression matrix of all detected genes in all cells. The data also needs to be checked to remove doublet cells. Scrublet (179), scds (180), DoubleFinder (181) were designed to identify doublets. Next, data normalization, integration, dimensionality reduction, and clustering should be performed, which could be realized by different functions implemented in Seurat (182). Cell clusters are further assigned “real” cell names by using computational tools, such as SingleR (183), CellAssign (184), AUCell (185). These cell names should be manually checked and will be used in the following advanced analysis. Most physiological and pathological processes are accompanied by transcriptional dynamics, which could be influenced by the pseudo-temporal ordering of single cells by using scRNA-seq data. Most commonly used expression trajectory inference tools include Monocle (186), Slingshot (187), PAGA (188), and DPT (189). Cell-cell interactions mediated by ligand-receptor complexes are critical in diverse biological processes. Investigation of context-dependent crosstalk of different cell types enables a deep understanding of specific physiological and pathological processes. ScRNA-seq data could also be used to infer cell-cell communications by using such computational tools as CellPhoneDB (190), CellChat (191), and NicheNet (192). Spatial transcriptomics techniques can assay cells in their native tissue context, which enables spatial characterization of transcriptional activities. Multiple computational tools have been developed to analyze spatial transcriptomics data, such as Seurat, BayesSpace (193), Giotto (194), stLearn (195). Integrative analysis of scRNA-seq and spatial transcriptomics data will help to precisely decode intercellular communications in specific tissue locations.

Here, we will summarize newly reported discoveries about inflammatory skin disease at single-cell resolution.

In 2018, Cheng *et al.* reported the first single-cell transcriptomics study of human epidermis from multiple anatomic sites and psoriasis-like skins. Intriguingly, high levels of previous reported inflammatory transcripts such as S100 transcripts and IFI27 levels in normal scalp were detected, suggesting a cause for the inflammation that often occurs at this site. While in foreskin keratinocytes, upregulation of proliferation-related transcripts was detected. In the psoriatic epidermis, enrichment of channel cells and mitotic subfraction, enhancement of inflammatory transcripts like *S100A7*, *S100A8*, *S100A9*, *IFI27*, *PI3* as well as CD1C^+^CD301A^+^ myeloid dendritic cell population were detected. This study provided a critical step toward epidermal development, differentiation, and inflammation (196). Psoriasis is known as an IL-17-driven inflammatory skin disease, in which autoantigen-induced CD8^+^ T cells have been identified as pathogenic drivers. By using scRNA-seq, a total of 11 transcriptionally diverse CD8^+^ T cell subsets in psoriatic and healthy skin were identified, including 2 non-exhausted Tc17 cell subsets. Besides, CXCL13, which achieved greater accuracy than IL-17A, was thought as a novel biomarker of psoriasis severity. This study uncovered the diverse landscape of CD8^+^ T cells in psoriatic and healthy skin (197). To uncover the expression of key phenotypic features of cells in both high fidelity and high throughput, Hughes et al. developed a massive parallel scRNA-seq, also called Seq-Well S3 (Second-Strand Synthesis) protocol to chart the transcriptional landscape of five human inflammatory skin diseases, including acne, alopecia areata, granuloma annulare, leprosy, and psoriasis (198). Over-representation of Tregs, dysfunctional NR4A1-expressing T cells, and senescent SESN3^+^ T cells were detected in psoriasis. Besides, IRF4^+^ cDC2 cluster that displays an elevated expression of CCL17, CCL22, and a population of fibroblasts that expressed CCL19 and BAFF were reported in psoriasis biopsies. Notably, a large population of shared signals among cell types and states in the tested five inflammatory diseases were identified, suggesting potentially common outputs between these diseases (198). He et al. reported the first scRNA-seq analysis of healthy, lesional, and nonlesional skin from AD patients. In this work, they detected a high expression of TH2 (IL-13) and TH22 (IL-22) T cells in AD. And identified a novel *COL6A5^+^COL18A1^+^* subpopulation inflammatory fibroblasts. These *COL6A5^+^COL18A1^+^* subpopulation fibroblasts expressed *CCL2* and *CCL9* cytokines and were unique to lesional AD. Another unique subpopulation to AD lesions identified in this work is *LAMP3* ^+^ DC, which expressed the *CCL19* receptor *CCR7*. These findings together revealed a potential role of fibroblast in cross-talk with DCs and T-cells (21). Rojahn et al. characterized the pathogenesis of AD on both transcriptomic and proteomic levels, by using suction blistering captured epidermal and biopsies samples. Comparing transcriptional profiles of key inflammatory pathways (such as TH2 pathways) were detected, but suction blistering was superior in cell-specific resolution for high-abundance transcripts (*i.e.* KRT1/KRT10, KRT16/KRT6A, S100A8/S100A9) (14). An elevated level of AD-typical cytokines such as IL-13 and IL-22 in TH2 and TH22 cells, as well as antimicrobial cytokines like IL-26 are which expressed in proliferating T cells and natural killer T cells, were detected. Gao et al. , evaluated the intrinsic and intercellular alterations of healthy donors and patients with psoriasis. They revealed that the evolutionally conserved epidermal keratinocytes and dermal mesenchymal cells could self-transform into immune active states *via* intensively evoking expression of major histocompatibility complex (MHC) genes during psoriasis. They uncovered the immunoregulatory axis from skin resident cells to immune cells (199). To generate the human skin cell atlas, single-cell technology combined with immunostaining *in situ* of human skin biopsies in early prenatal life, adulthood, and typical inflammatory skin diseases were characterized (176). In total, 34 cell states were identified in healthy human skin, with dynamic changes across embryonic, adult life, and upon perturbation during inflammatory skin diseases. In the view of the skin immune system, the dominant cells are lymphocytes and macrophages in first-trimester embryonic skin and clonal expansion of disease-associated lymphocytes in inflammatory diseases. In adult skin, two inferred trajectories for keratinocyte differentiation and the presence of endothelial cells were detected. Besides, augmented migratory DC signature was detected during the development of human thymus and in disease states. Taken together, this study revealed the dynamic nature of cutaneous homeostasis across the fetal development and immune-mediated inflammatory disorders (176).

## Concluding Remarks

The best strategy to characterize the pathological process and develop therapeutic targets of inflammatory skin diseases is to comparatively measure every key gene. However, it will take years to portray a large spectrum of genes by using traditional molecular techniques. The RNA-seq and single-cell transcriptomics technologies offer a great opportunity to extensively identify abnormalities in the pathological progression of inflammatory skin diseases. In this review, we discussed how transcriptomics data expedites significant findings in inflammatory skin diseases.

## Author Contributions

SL conceived and supervised this project. JW, ZF, TL, WH, and YW collected the data and literatures. ZF, SL, and WH draw the plots. SL and JW wrote the manuscript with comments from all listed authors. All authors contributed to the article and approved the submitted version.

## Funding

This study was supported by Shanghai General Hospital Startup Funding (02.06.01.20.06 and 02.06.02.21.01).

## Conflict of Interest

The authors declare that the research was conducted in the absence of any commercial or financial relationships that could be construed as a potential conflict of interest.

## Publisher’s Note

All claims expressed in this article are solely those of the authors and do not necessarily represent those of their affiliated organizations, or those of the publisher, the editors and the reviewers. Any product that may be evaluated in this article, or claim that may be made by its manufacturer, is not guaranteed or endorsed by the publisher.
